# Cyclophosphamide-induced seizures in a patient with neuropsychiatric systemic lupus erythematosus (NPSLE): A case report

**DOI:** 10.3389/fimmu.2023.1122629

**Published:** 2023-03-14

**Authors:** Lingshu Zhang, Ying Shi, Jingyao Zhang, Jing Wu, Wei Jiang

**Affiliations:** ^1^ Department of Rheumatology and Immunology, West China Hospital, Sichuan University, Chengdu, China; ^2^ Department of Integrated Traditional Chinese and Western Medicine, The Ninth People’s Hospital of Chongqing, Chongqing, China; ^3^ Core Facilities of West China Hospital, Sichuan University, Chengdu, China; ^4^ Internal Medicine Department, Xihua University Hospital, Chengdu, China

**Keywords:** neuropsychiatric lupus erythematosus, cyclophosphamide, seizure, side effect analysis, immunotherapy

## Abstract

Seizures are life-threatening complications of neuropsychiatric systemic lupus erythematosus (NPSLE) and are often associated with poor outcomes. Cyclophosphamide immunotherapy is the mainstay of NPSLE treatment. We report the unique case of a patient with NPSLE who developed seizures soon after her first and second doses of low-dose cyclophosphamide. The exact pathophysiological mechanism underlying cyclophosphamide-induced seizures is not well understood. However, this unusual drug-associated side effect of cyclophosphamide is thought to be due to the drug’s unique pharmacology. Clinicians should be aware of this complication to make a correct diagnosis and adjust the immunosuppressive regimens very carefully.

## Introduction

The nervous system is one of the major and frequent systems affected in the systemic lupus erythematosus (SLE) patient population. The manifestations of neuropsychiatric SLE (NPSLE) can be highly variable and fatal, and the American College of Rheumatology (ACR) proposed guidelines to define 19 neuropsychiatric syndromes seen in NPSLE, ranging from neurological to psychiatric symptoms, such as headache, seizures, cognitive dysfunction and cerebrovascular disease (1). The prevalence of seizure disorders is estimated to be between 7% and 40% in patients with SLE (1–5), and seizures are considered to be a major source of morbidity and mortality in the NPSLE population (6). The management of NPSLE remains unsatisfactory and is extremely challenging for clinicians. Cyclophosphamide, initially synthesized to target various malignant disorders, is also a potent agent widely and successfully utilized to treat autoimmune diseases, such as SLE. The main toxic effects of cyclophosphamide include bone marrow suppression, severe infections, digestive symptoms, gonadal toxicity and haemorrhagic cystitis (7). We present herein the unusual case of a patient with SLE who developed a generalized seizure and convulsions during cyclophosphamide treatment. Clinicians should be aware of this rare side effect of cyclophosphamide in patients with this complex condition.

## Case presentation

A 52-year-old woman was admitted to our hospital on 18 May 2022 with swollen hands and blurred vision in her left eye that had lasted for 6 months. She had been diagnosed with SLE and antiphospholipid syndrome in 2000, and her disease manifestations included lymphocytosis, lower limb numbness, decreased visual acuity and retinal artery occlusion. She took prednisone, warfarin and *Tripterygium wilfordii*, a traditional Chinese medicine, intermittently over the first few years after her diagnosis but had not taken any medication recently. She therefore came to our hospital for further diagnosis and treatment.

When she was transferred to our hospital, on physical examination, her vital signs were within normal limits, and heart, lung and abdomen examinations revealed normal findings. An examination of the extremities showed a scattered dark red rash on both lower limbs, and the fifth finger of the right hand was dark purple, but no lower limb oedema was observed. Neurological examination showed that the patient’s cognitive ability was decreased. Vision examination showed blurred left eye vision and her visual acuity in both eyes was decreased. She showed no abnormalities of muscle strength or a stiff neck with Kernig’s sign, and her deep tendon reflexes were symmetric. There were no signs of hair loss, photosensitivity, or oral ulcers. Laboratory test results showed a normal blood count. Stool and urine cultures were negative. The antinuclear antibody titre was 1/1000, with elevated IgG, anti-cardiolipin antibodies, lupus anticoagulant positivity and complement factor consumption (**Table 1**). Magnetic resonance imaging (MRI) of the head revealed multiple ischaemic foci and lacunar cerebral infarction in both cerebral hemispheres, the demyelination of white matter and mild brain atrophy.

**Table 1:** Inflammatory and rheumatologic laboratory findings.

Laboratory test	Result	Reference value
Antinuclear antibody	1:1000 speckled	<1:100
Rheumatoid factor	9.54IU/L	0-20
Histone antibodies, S	Negative	Negative
Anti-smith antibodies	Negative	Negative
Anti-dsDNA	Negative	Negative
Anti-RNP antibodies	Negative	Negative
SS-A	Negative	Negative
SS-B	Negative	Negative
Jo-1	Negative	Negative
SCL-70	Negative	Negative
C3	0.66	0.7-1.4
C4	0.2	0.1-0.4
Anti-CCP antibodies	3.5U/L	<5
ANCA	Negative	Negative
Complement C3	0.66	0.7-1.4
Complement C4	0.2	0.1-0.4
anti-β2GP1	>300AU/ml	<20
LA1/LA2	1.22	<1.2
IgG	13.48 g/L	8.6-17.4
IgA	1.25 g/L	1-4.2
IgM	1.41 g/L	0.5-2.8
Erythrocyte sedimentation rate	14mm/h	0–20
Hs-C reactive protein	1.9mol/L	0-5

RNP, ribonucleoprotein; CCP, cyclic citrullinated peptide; dsDNA, double-stranded DNA; ANCA, antineutrophil cytoplasmic antibodies; Anti-β2GP1, anti-beta 2 glycoprotein 1; LA, lupus anticoagulant.

Our patient was treated with methylprednisolone (MP; 40 mg intravenously once daily), warfarin (5 mg orally once a day), and hydroxychloroquine (200 mg orally twice a day), and she was started on cyclophosphamide 800 mg (600 mg/m2, approximately 15 mg/kg) on 19 May 2022. However, approximately 8-12 h after completing her first cyclophosphamide injection cycle to build up the dose, she vomited approximately 30 ml of blood and had a sudden, jerky movement of the limbs, along with cognitive dysfunction. Afterwards, she had a seizure episode with consciousness loss, frothing, and tongue biting associated with involuntary urination. She was immediately put on diazepam but still experienced three episodes of a tonic–clonic seizure. Her oxygen saturation gradually decreased and remained at 70% (8 L/m oxygen intake). Laboratory tests showed that routine blood counts and liver and kidney function parameters were within normal limits. She was urgently transferred to the intensive care unit for endotracheal intubation, with an invasive ventilator used for high-flow oxygen inhalation, and she received continuous sodium valproate injection for antiepileptic treatment as well as other symptomatic support treatment. MRI of the head found no additional abnormalities. She was discharged on the same continued medication of prednisone, warfarin, hydroxychloroquine, and sodium valproate with advice on regular follow-up and medication.

She was put on cyclophosphamide 800 mg on 11 July 2022. Again, she experienced a generalized tonic−clonic seizure. An episode of seizure with consciousness loss, frothing, and tongue biting was observed, which was managed conservatively with sodium valproate. Because the patient also experienced similar epileptic manifestations after the first injection of cyclophosphamide, the seizures were highly suspected to be related to cyclophosphamide, which was withdrawn.

The patient’s treatment was then adjusted as follows: MP was replaced with prednisone 40 mg orally once daily plus the immunosuppressant mycophenolate mofetil 0.75 g orally twice a day. Sodium valproate 5 mg was added for her symptoms. She was followed up in the outpatient department, and there was no occurrence of seizures thereafter.

## Discussion

SLE is a chronic multisystem autoimmune disorder that has a range of clinical presentations. The clinical manifestations of NPSLE affect the central and peripheral nervous systems, and the prevalence is estimated to be between 37% and 95% (8, 9). NPSLE is diverse and has a broad spectrum of at least 19 clinical neuropsychiatric syndromes proposed by the ACR (1). Among them, seizures represent a common manifestation and are present in 7% to 40% of SLE patients (mean 15%) (5). Seizures can be devastating manifestations of NPSLE in some patients and can even be fatal. To date, the distinct immune-pathogenetic mechanisms involved in NPSLE are incompletely understood, and key objective autoantibodies of clinical significance in the cerebrospinal fluid and/or serum are still awaiting discovery (10). There are no specific criteria to diagnose NPSLE. MRI is a widely adopted imaging modality and recognizes brain abnormalities in up to 75% of SLE patients (11, 12). However, despite advances in neuroimaging, limited key information and an inability to differentiate between NPSLE and non-NPSLE on conventional brain MRI remain (13).

Therapeutic management and the diagnostic process pose other challenges for clinicians. Controlled clinical trials provide limited guidance for the management of NPSLE. The European League Against Rheumatism published a consensus and developed recommendations, including general management involving high doses of corticosteroids in combination with an immunosuppressive treatment strategy, typically with cyclophosphamide, which shows superior efficacy to other (14, 15) synthetic disease-modifying antirheumatic drugs, such as mycophenolate, azathioprine and intrathecal methotrexate, in patients with NPSLE (16).

First synthesized for the treatment of cancer in 1958 (17), cyclophosphamide is also one of the most potent and widely used immunosuppressive agents in the treatment of rheumatic disorders and immune-mediated diseases. Cyclophosphamide is classified as an alkylating agent that is most active in the resting phase of the cell. This cytotoxic drug not only targets rapidly proliferating cancer cells but also can suppress the immune response in several pathways. The immunosuppressive properties of cyclophosphamide are mainly based on its interaction with DNA, by inducing DNA cross-linking and consequently inhibiting lymphocyte proliferation and reducing the production of pathogenic autoantibodies (18). The advantage of cyclophosphamide in the treatment of NPSLE is based on the active metabolites being highly protein-bound and distributed to all tissues, including the brain and cerebrospinal fluid. Following administration, reductions in the absolute numbers of both T cells and B cells are observed.

As this drug is cell-cycle specific, its major side effects are bone marrow suppression, gonadal toxicity, haemorrhagic cystitis, infectious complications and malignancies, and long-term high-dose use and a high cumulative dose of cyclophosphamide are the principal risk factors for side effects (19). Neurological symptoms are quite rare toxic effects of cyclophosphamide treatment. The administration of cyclophosphamide has been reported to induce posterior reversible encephalopathy syndrome in patients with anti-neutrophil cytoplasmic antibody associated vasculitis (20), lupus nephritis (21) and anti-glomerular basement membrane antibody glomerulonephritis (22, 23).

Our patient developed seizures after starting cyclophosphamide injections as a recommended therapy in the treatment of NPSLE. This is the first reported case of cyclophosphamide-induced seizures. The following mechanisms are suggested as the possible causes of seizures in our patient. First, cyclophosphamide may reduce the seizure threshold and alter neurotransmitter modulation in the brain. Similar to selective serotonin reuptake inhibitors, which can build up high levels of serotonin in the body and cause symptoms that can range from diarrhea to seizures (24, 25). Previous research found that cyclophosphamide has the capacity to induce 5-hydroxytryptamine release *via* the activation of enteric cholinergic neurons (26). The mechanism may be related to the excessive serotonergic activity in central nervous system (CNS) synapses leading to serotonin syndrome (serotonin toxicity), predisposing patients to seizures. Second, the blood−brain barrier (BBB) prevents most drugs from gaining access to the CNS parenchyma and limits the amount of drug reaching target pathological regions. The mechanisms underlying increased BBB permeability in NPSLE are not yet understood. However, the abnormalities of the BBB in NPSLE are sufficient for more cyclophosphamide to reach the CNS and cause seizure attacks. Another study demonstrated that astrocytic activation contributed to allodynia in a rat model of cyclophosphamide-induced cystitis (27). Activated neurons can produce numerous mediators, such as proinflammatory cytokines, that facilitate neuronal activity and synaptic plasticity. Furthermore, cyclophosphamide-induced hyponatremia and the associated decrease in blood sodium levels can directly cause abnormal discharge of cranial nerves. Several case reports have described severe acute hyponatraemic encephalopathy resulting from severe hyponatremia secondary to cyclophosphamide use that is thought to occur from drug-associated antidiuretic hormone release leading to free water retention (28, 29). However, our patient had no other symptoms, such as stupor, coma, or respiratory arrest, and her laboratory tests showed that her sodium level was within the normal limit before and after cyclophosphamide treatment.

It can be concluded that seizures represent a rare side effect of cyclophosphamide, and our case description suggests that clinicians should monitor patients carefully based on empirical judgement while managing NPSLE owing to the heterogeneous side effects of this drug.

## Conclusion

The present case report shows the possibility of seizure induction due to short-term use of cyclophosphamide therapy. Many knowledge gaps remain in our current understanding of the complex underlying pathogenic pathways in NPSLE. The fairly limited and nonspecific armamentarium of approved drugs remains a significant issue for NPSLE therapy. Physicians must always keep in mind that seizures can be induced while managing neurological symptoms in NPSLE patients.

## Data availability statement

The original contributions presented in the study are included in the article/supplementary material. Further inquiries can be directed to the corresponding author.

## Ethics statement

The studies involving human participants were reviewed and approved by The Ninth People’s Hospital of Chongqing. The patients/participants provided their written informed consent to participate in this study. Written informed consent was obtained from the individual(s) for the publication of any potentially identifiable images or data included in this article.

## Author contributions

LZ drafted the article. WJ and YS collected the clinical information. JW and JZ interpreted the data and WJ revised the article. All authors contributed to the article and approved the submitted version.
